# Treatment and survival of patients diagnosed with high-risk HR+/HER2− breast cancer in the Netherlands: a population-based retrospective cohort study ☆

**DOI:** 10.1016/j.esmoop.2024.103008

**Published:** 2024-04-26

**Authors:** S.W.M. Lammers, M. Meegdes, I.J.H. Vriens, A.C. Voogd, L. de Munck, T.J.A. van Nijnatten, K.B.M.I. Keymeulen, V.C.G. Tjan-Heijnen, S.M.E. Geurts

**Affiliations:** 1 Department of Medical Oncology, Maastricht University Medical Centre, GROW, Maastricht University, Maastricht; 2 Department of Epidemiology, Maastricht University, Maastricht; 3 Department of Research and Development, Netherlands Comprehensive Cancer Organisation, Utrecht; 4 Department of Radiology and Nuclear Medicine, Maastricht University Medical Centre, GROW, Maastricht; 5 Department of Surgery, Maastricht University Medical Centre, Maastricht, The Netherlands

**Keywords:** breast neoplasms, hormone receptor-positive, registries, survival, prognosis

## Abstract

**Background:**

Several factors may increase the risk of recurrence of patients diagnosed with hormone receptor-positive human epidermal growth factor receptor 2-negative (HR+/HER2−) breast cancer (BC). We aim to determine the proportion of patients with high-risk HR+/HER2− BC within the total HR+/HER2− BC cohort and compare their systemic treatments and survival rates with those of patients with low- and intermediate-risk HR+/HER2− BC and triple-negative (TN) BC.

**Patients and methods:**

Women diagnosed with nonmetastatic invasive HR+/HER2− BC and TNBC in the Netherlands between 2011 and 2019 were identified from the Netherlands Cancer Registry. Patients with HR+/HER2− BC were categorised according to risk profile, defined by nodal status, tumour size, and histological grade. High-risk HR+/HER2− BC was defined by either four or more positive lymph nodes or one to three positive lymph nodes with a tumour size of ≥5 cm or a histological grade 3 tumour. Overall survival (OS) and relative survival (RS) were calculated using the Kaplan–Meier and Pohar–Perme method.

**Results:**

In this study of 87 455 patients with HR+/HER2− BC, 44 078 (50%) patients were diagnosed with low risk, 28 452 (33%) with intermediate risk, and 11 285 (13%) with high-risk HR+/HER2− BC. In 3640 (4%) patients, the risk profile could not be defined. Endocrine therapy and chemotherapy were used in 38% and 7% of low-risk, 90% and 47% of intermediate-risk, and 94% and 73% of high-risk patients, respectively. The 10-year OS and RS rates were 84.1% [95% confidence interval (95% CI) 83.5% to 84.7%] and 98.7% (95% CI 97.3% to 99.4%) in low-risk, 75.1% (95% CI 74.2% to 76.0%) and 91.7% (95% CI 89.7% to 93.3%) in intermediate-risk, and 63.4% (95% CI 62.0% to 64.7%) and 72.3% (70.1% to 74.3%) in high-risk patients. The 10-year OS and RS rates of 12 689 patients with TNBC were 69.7% (95% CI 68.6% to 70.8%) and 79.1% (95% CI 77.0% to 80.9%), respectively.

**Conclusion:**

The poor prognosis of patients with high-risk HR+/HER2− BC highlights the need for a better acknowledgement of this subgroup and supports ongoing clinical trials aimed at optimising systemic therapy.

## Highlights

• High-risk HR+/HER2− BC was defined by ≥4 positive nodes or 1-3 positive nodes + a tumour size of ≥5 cm or a grade 3 tumour.
• One in eight women with HR+/HER2− BC were diagnosed with high-risk disease in the Netherlands between 2011 and 2019.
• In the real world, 94% and 73% of patients with high-risk HR+/HER2− BC received endocrine therapy and chemotherapy.
• The 10-year OS (63.4%) and RS rates (72.3%) of patients with high-risk HR+/HER2− BC were poor.
• Survival rates of patients with high-risk HR+/HER2− BC remained constant over the years.

## Introduction

Patients with hormone receptor-positive human epidermal growth factor receptor 2-negative (HR+/HER2−) breast cancer (BC) remain at risk of recurrence for many years after diagnosis.^1^ Several factors may increase the risk of recurrence, such as a larger tumour size, node-positive disease, and a higher histological grade.^1^, ^2^, ^3^ For example, a meta-analysis among women diagnosed with oestrogen receptor-positive (ER+) BC revealed that the risk of distant recurrence at 20 years after diagnosis increased from 22% in patients with no lymph nodes involved to as high as 52% in patients with four to nine lymph nodes involved.^1^

Several studies are currently investigating new treatment strategies in addition to standard endocrine therapy and chemotherapy in patients diagnosed with high-risk HR+/HER2− BC. While some studies are focussing on extending the duration of adjuvant endocrine therapy, others are directed at intensifying adjuvant endocrine therapy with new targeted therapies, such as cyclin-dependent kinase (CDK) 4/6 inhibitors or poly(adenosine diphosphate-ribose) polymerase (PARP) inhibitors.^4^, ^5^, ^6^, ^7^, ^8^, ^9^ However, although international guidelines provide systemic treatment recommendations by stage, histological grade, menopausal status, and subtype, little is known about how patients with high-risk HR+/HER2− BC are actually being treated in daily clinical practice.^10^, ^11^, ^12^ Furthermore, the long-term survival of this group of patients has only been reported for patients who participated in a clinical trial, thereby not representing real-world cases.^1^^,^^13^ A large American population-based study, however, showed that the short-term survival of patients diagnosed with high-risk HR+/HER2− BC may be comparable to the short-term survival of patients diagnosed with triple-negative (TN) BC.^14^

In this study, we therefore determine the proportion of patients with high-risk disease within a real-world cohort of patients diagnosed with nonmetastatic HR+/HER2− BC in the Netherlands between 2011 and 2019. In addition, we examine the systemic treatment use of patients diagnosed with low-, intermediate-, and high-risk HR+/HER2− BC and patients diagnosed with TNBC. Furthermore, we present the overall survival (OS) and relative survival (RS) rates of patients diagnosed with high-risk HR+/HER2− BC and compare these with those of patients diagnosed with low- and intermediate-risk HR+/HER2− BC and TNBC. We will also present results by period of diagnosis to determine whether the proportion, treatment, and survival rates of patients with high-risk HR+/HER2− BC changed over time.

## Patients and methods

### Patients

All patients (≥18 years) diagnosed with invasive HR+/HER2− BC and TNBC in the Netherlands between 2011 and 2019 were identified from the population-based Netherlands Cancer Registry (NCR). The NCR is maintained by trained registration clerks of the Netherlands Comprehensive Cancer Organisation, who retrospectively collect data regarding patient, tumour, and treatment characteristics from medical files. Survival status is updated annually through linkage with the Dutch Municipal Personal Records database. The last update for the current analysis was on 31 January 2022.

The following exclusion criteria were applied: diagnosis of an invasive BC within 10 years of the current BC, metastatic disease at diagnosis or within 3 months after diagnosis, no surgery of the primary tumour, diagnosis of a T4d tumour, and male sex. Patients with T4d tumours were excluded as we aimed to conform to the eligibility criteria of the monarchE trial.^15^ Male patients, whose clinicopathological characteristics and outcomes are known to differ from female patients, were excluded as we aimed to look at a homogeneous study population.^16^ Patients with a history of ductal carcinoma *in situ* or other invasive malignancies were eligible to represent real-world clinical practice. In the case of a synchronous bilateral invasive BC, the tumour with characteristics resulting in the poorest prognosis was selected. This was defined in the following order: the highest tumour–node–metastasis (TNM) stage, the highest histological grade, ER negativity, and HER2 negativity.^2^

### Definitions

In accordance with the Dutch BC guideline, tumours were considered HR+ when at least 10% of cells had positive nuclear staining of the ER and/or progesterone receptor (PR).^10^ Tumours were considered HER2− when an immunohistochemistry score of 0-1 or a negative fluorescence *in situ* hybridization result was present. When both the HR status and the HER2 status were negative, tumours were considered TN.

Patients with HR+/HER2− BC were categorised as low-, intermediate-, or high-risk (Supplementary Table S1, available at https://doi.org/10.1016/j.esmoop.2024.103008). The monarchE criteria were used to define high-risk disease: ≥4 positive lymph nodes or 1-3 positive lymph nodes and at least one of the following features: a tumour size of ≥5 cm or a histological grade 3 tumour.^15^ The Dutch guideline for the prescription of chemotherapy in patients with HR+/HER2− BC, which is largely in line with the St. Gallen criteria for systemic therapy in HR+/HER2− BC, was used to categorise all other patients as low- or intermediate-risk.^10^^,^^11^ In this analysis, patients without an indication for chemotherapy based on nodal status, tumour size, and histological grade were categorised as low-risk, whereas those with an indication for chemotherapy were categorised as intermediate-risk. Detailed information about the risk classification is provided in Supplementary Table S1, available at https://doi.org/10.1016/j.esmoop.2024.103008. The pathological tumour size was used when available. In patients with unknown pathological tumour size as well as patients who received neoadjuvant systemic therapy, the clinical tumour size was used when more advanced than the pathological tumour size. Tumours were considered node-positive based on either the clinical or pathological nodal status. The number of positive lymph nodes was based on the pathological nodal status. In clinically node-positive patients who received neoadjuvant systemic therapy, the positivity of one to three lymph nodes was assumed when more advanced than the pathological nodal status.

### Endpoints

Baseline characteristics and (neo)adjuvant systemic treatments were reported for the total, low-, intermediate-, and high-risk HR+/HER2− BC population and the total TNBC population. Systemic treatments were categorised as endocrine therapy, chemotherapy, endocrine therapy and chemotherapy, or no systemic therapy. OS was defined as the time from diagnosis of BC to death from any cause. RS, which functions as a surrogate measure for disease-specific survival in population-based studies where information on the cause of death is missing, was defined by the ratio between the OS of the study population and the expected survival of the Dutch population, taking into account age, sex, and calendar year.^17^

### Statistical analysis

The OS and RS rates of patients diagnosed with HR+/HER2− BC were analysed according to risk profile. Survival rates of patients with high-risk HR+/HER2− BC were also compared with those of patients diagnosed with TNBC, that is, patients who are generally considered ‘high-risk’. OS and RS were calculated with the Kaplan–Meier and Pohar–Perme method.^18^ Lifetables from Statistics Netherlands were used to determine the expected survival of the Dutch population. Differences between groups were assessed with the log-rank test and the Wald test. Patients alive at the last follow-up date were censored in all analyses.

In addition, baseline characteristics and systemic treatments were compared by period of diagnosis using the Cochran–Mantel–Haenszel test for trend for categorical variables and the Jonckheere–Terpstra test for trend for skewed continuous variables. Survival rates were also compared by period of diagnosis using multivariable Cox regression analyses for OS and Poisson regression analyses for RS.^19^ Missing values of confounders were imputed. The proportional hazards assumption and the presence of multicollinearity were tested.

Statistical analyses were carried out using SPSS (IBM Corp., New York, NY) and Stata (StataCorp LLC, College Station, TX). *P* values were two-sided and considered statistically significant at a value of ≤0.05.

## Results

### ***HR+/HER2***− ***BC***

This study included 87 455 women who were surgically treated for nonmetastatic invasive HR+/HER2− BC in the Netherlands between 2011 and 2019 (Supplementary Figure S1, available at https://doi.org/10.1016/j.esmoop.2024.103008). The median age at diagnosis was 62 years (range 20-101 years; Table 1). Most patients were diagnosed with a tumour size of ≤2 cm (65%), node-negative disease (66%), and a histological grade 1-2 tumour (84%).

**Table 1:** Baseline characteristics of patients diagnosed with nonmetastatic invasive HR+/HER2– breast cancer and triple-negative breast cancer in the Netherlands between 2011 and 2019, total and according to the risk profile

Characteristics	Total HR+/HER2− BC population (*n* = 87 455)	Low-risk HR+/HER2− BC population (*n* = 44 078)	Intermediate-risk HR+/HER2− BC population (*n* = 28 452)	High-risk HR+/HER2− BC population (*n* = 11 285)	Total TNBC population (*n* = 12 689)
Median age, years (range)	62 (20-101)	63 (22-101)	61 (20-101)	58 (20-95)	58 (22-98)
Age (years) at diagnosis, *n* (%)					
<40	2851 (3)	709 (2)	1155 (4)	734 (7)	1526 (12)
40-49	13 241 (15)	4789 (11)	4972 (18)	2618 (23)	2435 (19)
50-75	62 258 (71)	35 331 (80)	18 185 (64)	6474 (57)	7208 (57)
>75	9105 (10)	3249 (7)	4140 (15)	1459 (13)	1520 (12)
Tumour size (cm), *n* (%)					
≤2	56 443 (65)	42 523 (97)	9576 (34)	2690 (24)	5610 (44)
2.1-4.9	25 810 (30)	1555 (4)	17 741 (62)	4775 (42)	5991 (47)
≥5	5110 (6)	0 (0)	1135 (4)	3792 (34)	1069 (8)
Unknown, *n*	92	0	0	28	19
Lymph nodes, *n* (%)					
Negative	58 039 (66)	40 712 (92)	15 181 (53)	0 (0)	8481 (67)
1-3 positive	24 554 (28)	3366 (8)	13 271 (47)	6454 (57)	3327 (26)
4-9 positive	3235 (4)	0 (0)	0 (0)	3235 (29)	517 (4)
≥10 positive	1596 (2)	0 (0)	0 (0)	1596 (14)	359 (3)
Unknown, *n*	31	0	0	0	5
TNM stage^a^, *n* (%)					
Stage I	48 154 (55)	40 557 (92)	5803 (20)	512 (5)^b^	4636 (37)
Stage II	31 261 (36)	3438 (8)	22 206 (78)	3528 (31)	6358 (50)
Stage III	8004 (9)	76 (<1)	443 (2)	7245 (64)	1691 (13)
Unknown, *n*	36	7	0	0	4
Histological grade, *n* (%)					
Grade 1	25 298 (31)	22 049 (50)	2453 (9)	778 (8)	300 (3)
Grade 2	44 393 (54)	21 034 (48)	19 375 (68)	3951 (39)	2438 (21)
Grade 3	13 109 (16)	995 (2)	6624 (23)	5486 (54)	8748 (76)
Unknown, *n*	4655	0	0	1070	1203
Hormone receptor status, *n* (%)					
ER+/PR+	72 260 (83)	37 299 (85)	23 208 (82)	8886 (79)	NA
ER+/PR–	14 719 (17)	6675 (15)	5020 (18)	2292 (20)	NA
ER−/PR+	405 (1)	78 (<1)	202 (1)	93 (1)	NA
Unknown ER or PR status, *n*	71	26	22	14	NA
Histology, *n* (%)					
Ductal	66 835 (76)	34 873 (79)	20 953 (74)	8276 (73)	11 185 (88)
Lobular	15 859 (18)	6130 (14)	6350 (22)	2705 (24)	333 (3)
Other	4761 (5)	3075 (7)	1149 (4)	304 (3)	1171 (9)
Axillary staging, *n* (%)					
SNP	76 619 (88)	42 242 (96)	25 424 (89)	6168 (55)	9973 (79)
MARI	2176 (3)	81 (<1)	950 (3)	1004 (9)	626 (5)
ALND	13 838 (16)	1394 (3)	4538 (16)	7116 (63)	2730 (22)
None	2514 (3)	1385 (3)	660 (2)	177 (2)	462 (4)
Breast-conserving surgery, *n* (%)					
Yes	56 595 (65)	34 678 (79)	16 023 (56)	3771 (33)	7565 (60)
Radiotherapy, *n* (%)					
Yes	64 710 (74)	33 491 (76)	18 918 (67)	9707 (86)	9224 (73)

Percentages may not add up to 100% because of rounding.

ALND, axillary lymph node dissection; ER, oestrogen receptor; HR+/HER2− BC, hormone receptor-positive human epidermal growth factor receptor 2-negative breast cancer; MARI, marking axillary lymph nodes with radioactive iodine ^125^I seeds; NA, not applicable; PR, progesterone receptor; SNP, sentinel node procedure; TNBC, triple-negative breast cancer; TNM, tumour–node–metastasis.

a Tumours were categorised according to the TNM classification of malignant tumours which was valid at the time of diagnosis since only minor differences were present between the 7th and 8th editions. The pathological TNM stage was used when available. In patients with unknown pathological TNM stage as well as patients who received neoadjuvant systemic therapy, the clinical TNM stage was used.

b The high-risk population included 512 patients (5%) diagnosed with stage I disease as a result of including microscopic lymph node metastases when defining the number of lymph nodes.

In the total HR+/HER2− BC population, the performance of axillary lymph node dissections (ALNDs) decreased from 26% in 2011-2013 to 9% in 2017-2019 (Supplementary Table S2, available at https://doi.org/10.1016/j.esmoop.2024.103008). In addition, the percentage of patients with four or more positive lymph nodes (8% versus 4%) or a histological grade 3 tumour (18% versus 14%) decreased, whereas the percentage of patients with a tumour size of ≥5 cm (5% versus 6%) remained more or less constant.

### Risk classification

**Figure 1:**
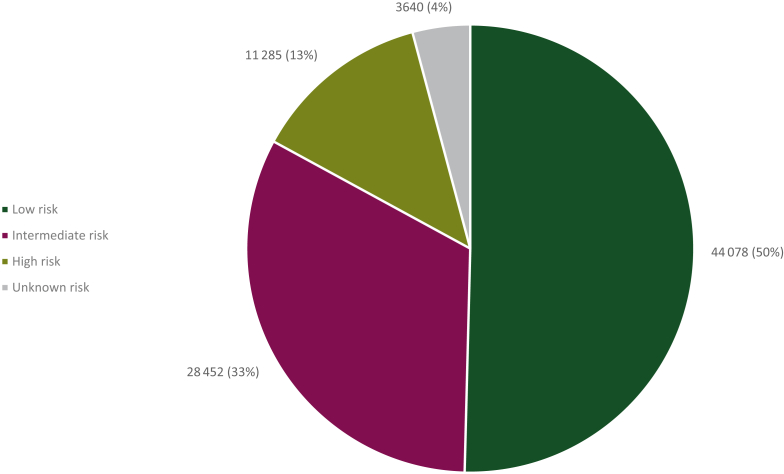
Risk classification of patients diagnosed with nonmetastatic invasive hormone receptor-positive human epidermal growth factor receptor 2-negative (HR+/HER2−) breast cancer in the Netherlands between 2011 and 2019.

Of 87 455 patients with HR+/HER2− BC, 44 078 (50%) were classified as low-risk, 28 452 (33%) were classified as intermediate-risk, and 11 285 (13%) were classified as high-risk (Figure 1). In 3640 (4%) patients, the risk profile could not be defined.

When compared with low-risk patients, high-risk patients were younger (median age 58 versus 63 years), more frequently diagnosed with a lobular tumour (24% versus 14%), and less frequently diagnosed with both an ER+ and PR+ tumour (79% versus 85%; Table 1). As expected, tumour size, number of positive lymph nodes, TNM stage, and histological grade increased with an increasing risk classification. The performance of ALNDs also increased with an increasing risk classification: 63% of high-risk patients received an ALND, whereas only 3% of low-risk patients received an ALND.

The percentage of high-risk patients decreased from 15% in 2011-2013 to 11% in 2017-2019 (Supplementary Figure S2, available at https://doi.org/10.1016/j.esmoop.2024.103008). When baseline characteristics of high-risk patients were compared by period of diagnosis, a significant decrease in ALNDs was observed: from 82% in 2011-2013 to 45% in 2017-2019 (Supplementary Table S3, available at https://doi.org/10.1016/j.esmoop.2024.103008). The percentage of high-risk patients with four or more positive lymph nodes also decreased (52% in 2011-2013 versus 34% in 2017-2019). The proportion of high-risk patients with a histological grade 3 tumour (57% versus 51%) or a tumour size of ≥5 cm (27% versus 40%) changed slightly as well.

### Systemic therapy use

Within the HR+/HER2− BC population, 38% of low-risk, 90% of intermediate-risk, and 94% of high-risk patients received endocrine therapy (Table 2). Chemotherapy was prescribed in 7%, 47%, and 73% of patients, respectively. In high-risk patients, the use of chemotherapy was strongly associated with age: 93% of patients aged <65 years versus 34% of patients aged ≥65 years received chemotherapy. From 2011 to 2019, the proportion of high-risk patients receiving endocrine therapy or chemotherapy remained stable (Supplementary Table S4, available at https://doi.org/10.1016/j.esmoop.2024.103008). However, the use of neoadjuvant chemotherapy increased from 20% in 2011-2013 to 42% in 2017-2019, whereas the use of adjuvant chemotherapy decreased from 56% to 34%.

**Table 2:** Systemic treatment choices in patients diagnosed with HR+/HER2− breast cancer and triple-negative breast cancer, total and according to risk profile

Systemic treatment	Total HR+/HER2− BC population (*n* = 87 455)	Low-risk HR+/HER2− BC population (*n* = 44 078)	Intermediate-risk HR+/HER2− BC population (*n* = 28 452)	High-risk HR+/HER2− BC population (*n* = 11 285)	Total TNBC population (*n* = 12 689)
Endocrine therapy, *n* (%)	55 439 (63)	16 577 (38)	25 681 (90)	10 584 (94)	NA
Neoadjuvant	2725 (3)	314 (1)	1418 (5)	599 (5)	NA
Adjuvant	55 300 (63)	16 535 (38)	25 615 (90)	10 568 (94)	NA
Chemotherapy, *n* (%)	26 737 (31)	3130 (7)	13 343 (47)	8222 (73)	8809 (69)
Neoadjuvant	9321 (11)	233 (1)	3770 (13)	3544 (31)	4445 (35)
Adjuvant	17 900 (21)	2901 (7)	9711 (34)	4985 (44)	5298 (42)
Endocrine therapy and chemotherapy, *n* (%)	25 316 (29)	2960 (7)	12 620 (44)	7812 (69)	NA
No systemic therapy, *n* (%)	30 593 (35)	27 329 (62)	2048 (7)	291 (3)	3836 (30)

HR+/HER2− BC, hormone receptor-positive human epidermal growth factor receptor 2-negative breast cancer; NA, not applicable; TNBC, triple-negative breast cancer.

### Survival

The median follow-up period was 6.5 years (interquartile range 4.3-8.7), during which 10 864 patients with HR+/HER2− BC had died. The 10-year OS and RS rates of the total HR+/HER2− BC population were 78.3% [95% confidence interval (CI) 77.8% to 78.7%] and 92.4% (95% CI 91.5% to 93.2%), respectively. In multivariable analysis, when compared with patients diagnosed in 2011-2013, statistically significant improvements in the OS of patients diagnosed in 2014-2016 [hazard ratio (HR) 0.95, 95% CI 0.91-1.00] and patients diagnosed in 2017-2019 (HR 0.90, 95% CI 0.84-0.96) were observed (Supplementary Figure S3A and Table S5, available at https://doi.org/10.1016/j.esmoop.2024.103008). However, in the total HR+/HER2− BC population, no difference in RS was observed over the years (Supplementary Figure S3B and Table S6, available at https://doi.org/10.1016/j.esmoop.2024.103008).

**Figure 2:**
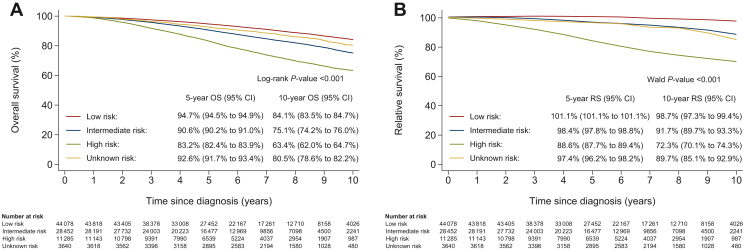
**(A) Overall survival (OS) and (B) relative survival (RS) of patients diagnosed with nonmetastatic invasive hormone receptor-positive human epidermal growth factor receptor 2-negative (HR+/HER2−) breast cancer from the date of diagnosis, according topredefined risk profile.** CI, confidence interval.

In the survival analyses by risk profile, the 10-year OS and RS rates were 84.1% (95% CI 83.5% to 84.7%) and 98.7% (95% CI 97.3% to 99.4%) in low-risk, 75.1% (95% CI 74.2% to 76.0%) and 91.7% (95% CI 89.7% to 93.3%) in intermediate-risk, and 63.4% (95% CI 62.0% to 64.7%) and 72.3% (70.1% to 74.3%) in high-risk patients, respectively (Figure 2A and B). Over the years, no difference in the OS and RS of patients diagnosed with high-risk HR+/HER2− BC was observed (Supplementary Figure S4A and B, Tables S7 and S8, available at https://doi.org/10.1016/j.esmoop.2024.103008).

### TNBC

This study included 12 689 women who were surgically treated for nonmetastatic invasive TNBC in the Netherlands between 2011 and 2019 (Supplementary Figure S1, available at https://doi.org/10.1016/j.esmoop.2024.103008). When compared with patients diagnosed with high-risk HR+/HER2− BC, patients diagnosed with TNBC less frequently had a tumour size of ≥5 cm (8% versus 34%) and node-positive disease (33% versus 100%; Table 1). However, the percentage of patients with a histological grade 3 tumour was higher in the TNBC group (76% versus 54%). Overall, 69% of patients diagnosed with TNBC received chemotherapy (Table 2). As was the case for patients diagnosed with high-risk HR+/HER2− BC, the use of chemotherapy was strongly associated with age: 87% of patients aged <65 years versus 35% of patients aged ≥65 years received chemotherapy.

**Figure 3:**
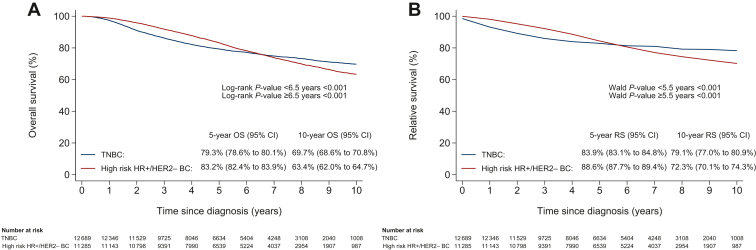
**(A) Overall survival (OS) and (B) relative survival (RS) from the date of diagnosisofpatients diagnosed with triple-negative breast cancer (TNBC) and high-risk hormone receptor-positive human epidermal growth factor receptor 2-negative (HR+/HER2−) breast cancer.** CI, confidence interval.

The median follow-up period was 6.5 years (interquartile range 4.2-8.8), during which 2921 patients diagnosed with TNBC had died. While 5-year OS and RS rates of patients diagnosed with TNBC (79.3%, 95% CI 78.6% to 80.1% and 83.9%, 95% CI 83.1% to 84.8%, respectively) were significantly worse than those of patients diagnosed with high-risk HR+/HER2− BC (83.2%, 95% CI 82.4% to 83.9% and 88.6%, 95% CI 87.7% to 89.4%, respectively), the opposite was observed for the 10-year OS and RS rates (Figure 3A and B). Specifically, the 10-year OS and RS rates of patients diagnosed with TNBC (69.7%, 95% CI 68.6% to 70.8% and 79.1%, 95% CI 77.0% to 80.9%, respectively) were higher than those of patients diagnosed with high-risk HR+/HER2− BC (63.4%, 95% CI 62.0% to 64.7% and 72.3%, 95% CI 70.1% to 74.3%, respectively).

## Discussion

In this large population-based study of 87 455 patients surgically treated for nonmetastatic invasive HR+/HER2− BC between 2011 and 2019 in the Netherlands, we showed that 13% of patients were diagnosed with high-risk disease. The prognosis of patients with high-risk HR+/HER2− BC was poor, as depicted by a 10-year OS rate of 63.4% and a 10-year RS rate of 72.3%. We showed that survival outcomes of patients with high-risk HR+/HER2− BC were significantly worse than those of patients with low- and intermediate-risk HR+/HER2− BC, and those of patients with TNBC. These data suggest that the current real-world use of endocrine therapy (94%) and chemotherapy (73%) is insufficient for patients with high-risk HR+/HER2− BC.

Recently, several studies have used the monarchE criteria to categorise patients with HR+/HER2− BC as ‘high-risk’.^13^^,^^14^^,^^20^^,^^21^ Most of these studies reported percentages similar to that of high-risk patients (13%) in our study. In a large American population-based study [Surveillance, Epidemiology, and End Results (SEER) database], for example, 12% of 238 222 patients diagnosed with HR+/HER2− BC in 2010-2015 were categorised as high-risk.^14^ In another American study (Flatiron Health database), 14% of 4028 patients diagnosed with HR+/HER2− BC in 2011-2020 were classified as high-risk.^20^ The proportion of high-risk patients may further increase when a high Ki-67 score (≥20%) is added as an additional risk factor.^22^ However, Ki-67 scores are currently not registered in the NCR and therefore not included in our risk classification.

In our study, the percentage of patients diagnosed with high-risk HR+/HER2− BC slightly decreased from 15% in 2011-2013 to 11% in 2017-2019. There are several potential explanations for this decrease. First, in the total HR+/HER2− BC study population, the simultaneous decrease in ALNDs from 26% in 2011-2013 to 9% in 2017-2019 may be an important factor to consider, as this decrease likely contributed to the decrease of patients with four or more lymph nodes involved. A second explanation for the decrease in high-risk patients over the years may be the increased use of ^18^F-fluorodeoxyglucose (^18^F-FDG)–positron emission tomography/computed tomography (PET/CT) imaging as an initial staging modality in recent years.^23^ Several studies have shown that, when compared with conventional staging modalities, ^18^F-FDG–PET/CT imaging has a higher accuracy for diagnosing distant metastases.^24^, ^25^, ^26^, ^27^ Some of the patients who would in the earlier years be classified as high-risk may now be excluded from this study because of the diagnosis of metastatic disease. In addition, the increased use of neoadjuvant chemotherapy may have contributed to the decrease in high-risk patients over the years. It is also important to recognise the influence of population-based BC screening on determining the percentage of high-risk patients. Screen-detected cancers are less likely to present at an advanced stage.^28^ The percentage of high-risk patients is therefore expected to be much higher in developing countries, which have not yet implemented BC screening.

As far as we know, we are the first to report 10-year survival outcomes of patients diagnosed with high-risk HR+/HER2− BC in a real-world setting. The 10-year RS rate (72.3%) is of particular concern, as this percentage indicates that one in four women with high-risk HR+/HER2− BC will die from BC within 10 years after diagnosis. The unchanged, equally worse, survival outcomes of patients diagnosed with high-risk HR+/HER2− BC over the years are also of particular concern, suggesting that we are not making any improvements in the treatment of patients with high-risk HR+/HER2− BC. The lack of an improvement in the prognosis of patients with high-risk HR+/HER2− BC may, however, also be related to counteracting stage migration, as a result of the decrease in ALNDs and increased use of ^18^F-FDG–PET/CT imaging over the years, which we discussed earlier.^24^, ^25^, ^26^, ^27^ The aforementioned SEER study is the only other study that provided 5-year (relative) survival data of patients with high-risk HR+/HER2− BC in a real-world setting.^14^ Remarkably, their 5-year BC-specific mortality (BCSM) rate (16.5%) was slightly higher than that of our high-risk population (i.e. 11.4%).^14^ Obviously, this difference in short-term BCSM rates might be related to differences in patient and tumour characteristics, that is, in the American high-risk cohort, patients were more frequently diagnosed with stage 3 disease (69% versus 64%) and four or more positive lymph nodes (53% versus 43%).^14^ Variations in (access to) locoregional and systemic therapy may also account for the difference in short-term BCSM rates. Unfortunately, the American study did not provide information about prescribed treatments, and it is unknown whether all high-risk patients received curative surgery or not, which was a key inclusion criterion for our study.

Long-term survival rates of our high-risk HR+/HER2− BC population were significantly worse than those of our TNBC population. It is well known that patients with TNBC, when compared with other BC subtypes, experience an increased risk of recurrence during the first 5 years after diagnosis.^29^^,^^30^ Our 5-year results show a similar pattern, that is, 5-year OS and RS rates were lower in the TNBC population (79.3% and 83.9%) versus the high-risk HR+/HER2− BC population (83.2% and 88.6%). In the aforementioned SEER study, the 5-year BCSM was also higher in the TNBC population (18.5%) versus the high-risk HR+/HER2− BC population (16.5%).^14^ However, after the first 5 years, the risk of recurrence of patients with TNBC decreases significantly.^29^^,^^31^ By contrast, the risk of recurrence of patients with HR+/HER2− BC remains elevated for many years after diagnosis and may result in a lifelong risk of recurrence.^1^ Our results do, therefore, not come as a surprise, especially when considering that patients with TNBC were included irrespective of risk profile. Patients with TNBC were, for example, less frequently diagnosed with a tumour size of ≥5 cm (8% versus 34%) or node-positive disease (33% versus 100%) when compared with patients with high-risk HR+/HER2− BC. These comparisons do, however, help to put results into perspective and clearly demonstrate that the long-term prognosis of patients with high-risk HR+/HER2− BC remains disappointing. A critical reflection on their current treatment patterns seems appropriate.

In our high-risk HR+/HER2− BC cohort, 94% of patients received endocrine therapy and 73% of patients received chemotherapy. These percentages of systemic therapy remained stable over the years. The use of endocrine therapy was high and in line with what would be expected in the real world, in which a percentage of 100% is not realistic because of patient preferences and comorbidities. The low use of chemotherapy is noticeable and requires attention. There are several potential explanations for the low use of chemotherapy. Age and comorbidities may have contraindicated chemotherapy use. We, for example, observed that only 34% of high-risk patients aged ≥65 years received chemotherapy. The low use of chemotherapy among older patients with BC has been observed earlier and is not very surprising because the Dutch BC guideline is cautious about the use of chemotherapy in older patients.^10^^,^^32^, ^33^, ^34^ Patients may also refuse chemotherapy. The use of chemotherapy in our cohort of high-risk patients was similar to that of the American real-world study (Flatiron Health database) and even higher than that of a German single-centre study, in which 68% and 51% of patients received chemotherapy, respectively.^20^^,^^22^ The use of chemotherapy was, however, way lower than the use of chemotherapy in clinical trials (88%-100%).^8^^,^^13^^,^^15^ This difference in chemotherapy use should be acknowledged when evaluating the benefit of new targeted therapies in the real world.

The optimal treatment of patients with high-risk HR+/HER2− BC is currently under debate. A potential strategy to decrease the recurrence risk in high-risk patients is extending the duration of adjuvant endocrine therapy.^4^, ^5^, ^6^ In the DATA trial, for example, postmenopausal women diagnosed with ER+/PR+, node-positive tumours of larger size (≥pT2) experienced major improvements in disease-free survival (DFS; HR 0.64, 95% CI 0.47-0.88) when receiving 6 versus 3 years of anastrozole after 2-3 years of adjuvant tamoxifen.^5^ Another promising new treatment option for patients with high-risk HR+/HER2− BC is intensifying adjuvant endocrine therapy with a CDK 4/6 inhibitor.^7^^,^^8^ In the monarchE trial, for example, patients receiving at least 5 years of endocrine therapy in combination with 2 years of abemaciclib experienced a statistically significant reduction in invasive DFS (HR 0.66, 95% CI 0.58-0.76) and distant relapse-free survival (HR 0.66, 95% CI 0.57-0.77) when compared with patients receiving endocrine monotherapy.^7^ Similar results were observed in the NATALEE trial, which evaluated the use of 3 years of ribociclib in intermediate- and high-risk patients assigned to at least 5 years of treatment with an aromatase inhibitor.^8^ In addition, the OLYMPIA trial showed that patients with high-risk HER2− disease and a germline BRCA1 or BRCA2 mutation may benefit from adjuvant treatment with olaparib, a PARP inhibitor, after completing local treatment and (neo)adjuvant chemotherapy.^9^ Several trials are currently also investigating the effectiveness of oral selective ER degraders in the adjuvant setting.^35^ All these treatments seem promising for patients with high-risk HR+/HER2− BC.

One of the major strengths of our study is the use of a large population-based registry. The NCR includes all new cases of BC in the Netherlands, resulting in high completeness and the elimination of selection bias. Our study also has certain limitations. First, a misclassification of patients according to risk profile may have occurred. While both clinical and pathological nodal status were used to distinguish between node-positive and node-negative disease, the number of positive lymph nodes was exclusively based on the pathological status. This may have resulted in an underestimation of the number of positive lymph nodes in patients who received neoadjuvant systemic therapy. In addition, our study lacked detailed information on systemic treatment, disease recurrence, and cause of death, because these data are not registered in the NCR. The lack of disease-specific outcomes was, however, overcome by including RS as a surrogate measure for disease-specific survival.^17^ Furthermore, as information on vital status was available until 31 January 2022, long-term survival outcomes reflect those of patients diagnosed in the earlier years of the study period (2011-2019). The impact on the study results is nonetheless expected to be small, as we observed that OS and RS of patients diagnosed with high-risk HR+/HER2− BC did not differ by period of diagnosis.

In conclusion, one in eight women with HR+/HER2− BC were diagnosed with high-risk disease in the Netherlands between 2011 and 2019. The 10-year OS (63.4%) and RS (72.3%) rates of patients with high-risk HR+/HER2− BC were poor and constant over the years, suggesting that no improvements in the treatment of patients with high-risk HR+/HER2− BC have been made over the last decade. The real-world use of chemotherapy (73%) in patients with high-risk HR+/HER2− BC was low and requires attention. Awareness of the poor prognosis of patients with high-risk HR+/HER2− BC may help to increase the use of chemotherapy and support the implementation of new systemic therapies and ongoing clinical trials aimed at optimising systemic therapy for this subgroup of patients.
