# Supplementary material for: Treatment and survival of patients diagnosed with high-risk HR+/HER2− breast cancer in the Netherlands: a population-based retrospective cohort study
Source: ESMO Open. 2024 Apr 26;9(5):103008. doi: 10.1016/j.esmoop.2024.103008 (PMC11067336; doi:10.1016/j.esmoop.2024.103008)
Supplement: Supplementary data [file mmc1.pdf]

Supplementary appendix

Supplement to: Lammers S.W.M., Meegdes M., Vriens I.J.H. et al. Treatment and survival of patients diagnosed with high-risk HR+/HER2- breast cancer in the Netherlands: a population-based retrospective cohort study.

Corresponding author:
Sandra M.E. Geurts, PhD
ORCID ID: 0000-0002-4265-9968
Department of Medical Oncology, Maastricht University Medical Centre,
P.O. Box: 5800, 6202 AZ Maastricht, the Netherlands
Email: sandra.geurts@mumc.nl
Phone: +31433877025

Table of contents

Supplementary Table 1. Risk classification 3

Supplementary Figure 1. Flowchart of included patients 4

Supplementary Table 2. Baseline characteristics of patients diagnosed with and surgically treated for non-metastatic invasive HR+/HER2- breast cancer in the Netherlands between 2011 and 2019 by period of diagnosis (N (%)) 5

Supplementary Figure 2. Number and proportion of patients diagnosed with and surgically treated for non-metastatic invasive HR+/HER2- breast cancer in the Netherlands between 2011 and 2019 according to risk profile, total and per 3-year incidence period 7

Supplementary Table 3. Baseline characteristics of patients diagnosed with high-risk HR+/HER2- breast cancer by period of diagnosis (N (%)) 8

Supplementary Table 4. Systemic treatment choices in patients diagnosed with high-risk HR+/HER2- breast cancer by period of diagnosis (N (%)) 10

Supplementary Figure 3. Overall survival (A) and relative survival (B) of all patients diagnosed with and surgically treated for non-metastatic invasive HR+/HER2- breast cancer from date of diagnosis, per 3-year incidence period 11

Supplementary Table 5. Univariable and multivariable analyses of overall survival in all patients diagnosed with HR+/HER2- breast cancer 12

Supplementary Table 6. Univariable and multivariable analyses of relative survival in all patients diagnosed with HR+/HER2- breast cancer 13

Supplementary Figure 4. Overall survival (A) and relative survival (B) of patients diagnosed with high-risk HR+/HER2- breast cancer from date of diagnosis, per 3-year incidence period 14

Supplementary Table 7. Univariable and multivariable analyses of overall survival in patients diagnosed with high-risk HR+/HER2- breast cancer 15

Supplementary Table 8. Univariable and multivariable analyses of relative survival in patients diagnosed with high-risk HR+/HER2- breast cancer 16

Supplementary Table 1. Risk classification

Histological grade	Tumour size[a]	Node-negative	1-3 positive lymph nodes[b]	≥4 positive lymph nodes[b]
Grade 1	≤2 cm	Low-risk	Low-risk	High-risk
	2.1 – 3 cm	Low-risk[c]	Intermediate-risk	High-risk
	3.1 – 4.9 cm	Intermediate-risk	Intermediate-risk	High-risk
	≥5 cm	Intermediate-risk	High-risk	High-risk
	Unknown	Unknown-risk	Unknown-risk	High-risk
Grade 2	≤2 cm	Low-risk[c]	Intermediate-risk	High-risk
	2.1 – 4.9 cm	Intermediate-risk	Intermediate-risk	High-risk
	≥5 cm	Intermediate-risk	High-risk	High-risk
	Unknown	Unknown-risk	Unknown-risk	High-risk
Grade 3	≤1 cm	Low-risk	High-risk	High-risk
	1.1 – 2 cm	Intermediate-risk	High-risk	High-risk
	2.1 – 4.9 cm	Intermediate-risk	High-risk	High-risk
	≥5 cm	Intermediate-risk	High-risk	High-risk
	Unknown	Unknown-risk	High-risk	High-risk
Unknown	≤2 cm	Unknown-risk	Unknown-risk	High-risk
	2.1 - 4.9 cm	Unknown-risk	Unknown-risk	High-risk
	≥5 cm	Unknown-risk	High-risk	High-risk
	Unknown	Unknown-risk	Unknown-risk	High-risk

[a]The pathological tumour size was used when available. In patients with unknown pathological tumour size as well as patients who received neoadjuvant systemic therapy, the clinical tumour size was used when more advanced than the pathological tumour size.
[b]Tumours were considered node-positive based on either the clinical or pathological nodal status. The number of positive lymph nodes was based on the pathological nodal status. In clinically node-positive patients who received neoadjuvant systemic therapy, the positivity of one to three lymph nodes was assumed when more advanced than the pathological nodal status.
[c]In patients aged younger than 35 years, node-negative tumours were considered intermediate-risk when having the following features:
- Grade 1 tumour of more than 2 cm in size;
- Grade 2 tumour of more than 1 cm in size.

Supplementary Figure 1. Flowchart of included patients

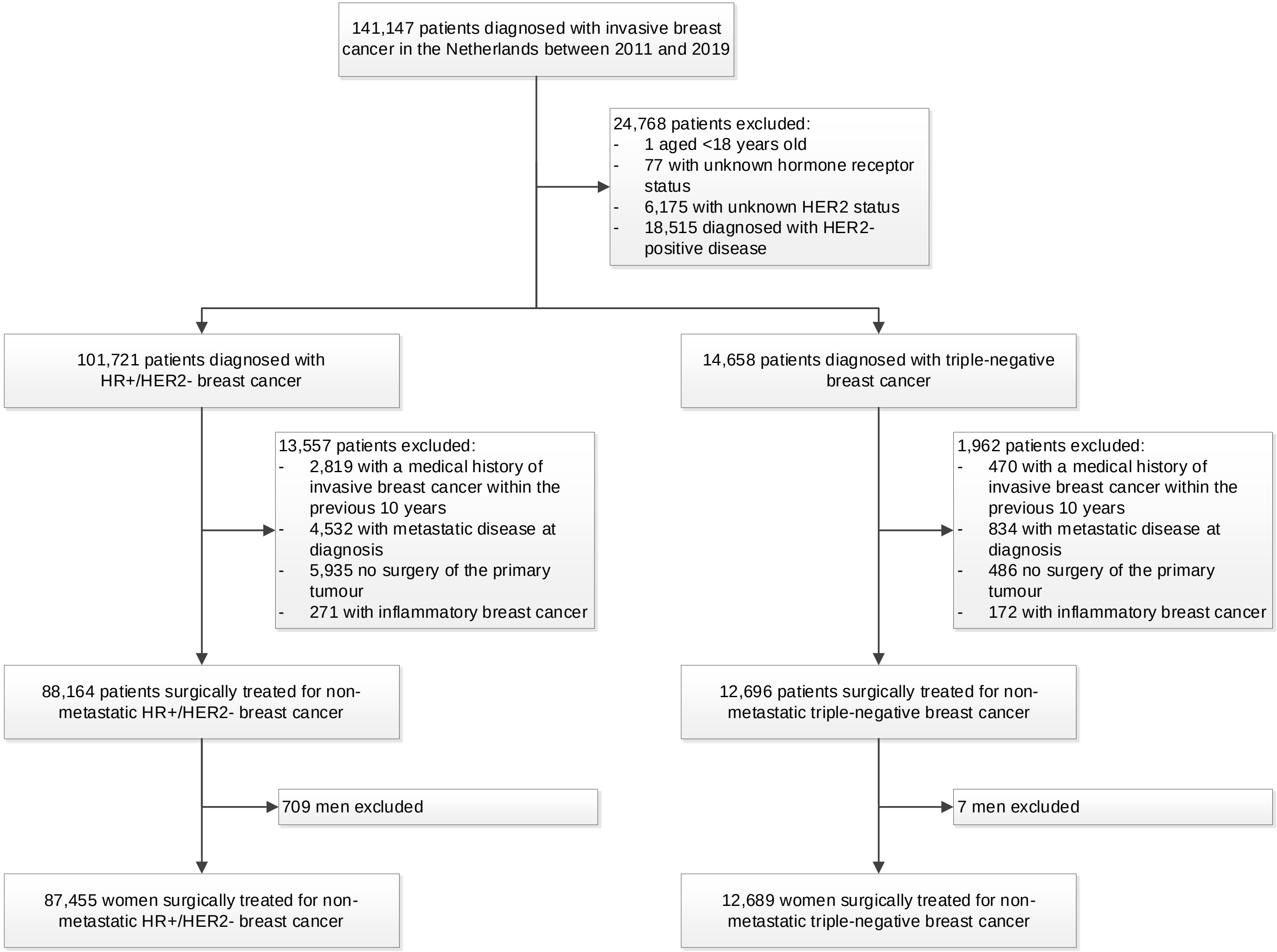

Supplementary Table 2. Baseline characteristics of patients diagnosed with and surgically treated for non-metastatic invasive HR+/HER2- breast cancer in the Netherlands between 2011 and 2019 by period of diagnosis (N (%))

	2011 – 2013 (n = 28,464)	2014 – 2016 (n = 29,190)	2017 – 2019 (n = 29,801)	P for trend
Median age, years (range)	61 (21-97)	62 (20-101)	62 (21-101)	<0.001
Age at diagnosis				0.08
<40 years	854 (3)	978 (3)	1,019 (3)	
40 – 49 years	4,659 (16)	4,234 (15)	4,348 (15)	
50 – 75 years	19,934 (70)	21,030 (72)	21,294 (72)	
>75 years	3,017 (11)	2,948 (10)	3,140 (11)	
Tumour size				<0.001
≤2 cm	18,534 (65)	18,943 (65)	18,966 (64)	
2.1-4.9 cm	8,398 (30)	8,487 (29)	8,925 (30)	
≥5 cm	1,480 (5)	1,738 (6)	1,892 (6)	
Unknown	52	22	18	
Lymph nodes				<0.001
Negative	18,400 (65)	19,359 (66)	20,280 (68)	
1-3 positive	7,863 (28)	8,353 (29)	8,338 (28)	
4-9 positive	1,453 (5)	988 (3)	794 (3)	
≥10 positive	731 (3)	485 (2)	380 (1)	
Unknown	17	5	9	
TNM stage[a]				<0.001
Stage I	15,474 (54)	16,231 (56)	16,449 (55)	
Stage II	9,997 (35)	10,375 (36)	10,889 (37)	
Stage III	2,974 (10)	2,575 (9)	2,455 (8)	
Unknown	19	9	8	
Histological grade				<0.001
Grade 1	8,106 (31)	8,399 (31)	8,793 (30)	
Grade 2	13,348 (51)	14,865 (54)	16,180 (55)	
Grade 3	4,614 (18)	4,267 (16)	4,228(15)	
Unknown	2,396	1,659	600	
Hormone receptor status				0.36
ER+/PR+	23,396 (82)	24,203 (83)	24,661 (83)	
ER+/PR-	4,900 (17)	4,822 (17)	4,997 (17)	
ER-/PR+	110 (<1)	157 (1)	138 (1)	
Unknown ER or PR status	58	8	5	
Histology				<0.001
Ductal	22,007 (77)	22,258 (76)	22,570 (76)	
Lobular	4,878 (17)	5,309 (18)	5,672 (19)	
Other	1,579 (6)	1,623 (6)	1,559 (5)	
Axillary staging				
SNP	24,428 (86)	25,828 (89)	26,363 (89)	<0.001
MARI	0 (0)	505 (2)	1,671 (6)	<0.001
ALND	7,326 (26)	3,847 (13)	2,665 (9)	<0.001
None	554 (2)	782 (3)	1,178 (4)	<0.001
Breast-conserving surgery				<0.001
Yes	17,295 (61)	18,856 (65)	20,444 (69)	
Radiotherapy				<0.001
Yes	20,181 (71)	22,084 (76)	22,445 (75)	
Endocrine therapy				<0.001
Yes	18,929 (67)	18,708 (64)	17,802 (60)	

Chemotherapy					<0.001
	Yes	10,371 (36)	9,207 (32)	7,159 (24)	

Abbreviations: ALND = axillary lymph node dissection; ER = oestrogen receptor; MARI = marking axillary lymph nodes with radioactive iodine ^{125}I seeds; PR = progesterone receptor; SNP = sentinel node procedure; TNM = tumour node metastasis.

Percentages may not add up to 100% because of rounding.

[a]Tumours were categorised according to the TNM classification of malignant tumours which was valid at the time of diagnosis, since only minor differences were present between the 7th or 8th edition. The pathological TNM stage was used when available. In patients with unknown pathological TNM stage as well as patients who received neoadjuvant systemic therapy, the clinical TNM stage was used.

Supplementary Figure 2. Number and proportion of patients diagnosed with and surgically treated for non-metastatic invasive HR+/HER2- breast cancer in the Netherlands between 2011 and 2019 according to risk profile, total and per 3-year incidence period

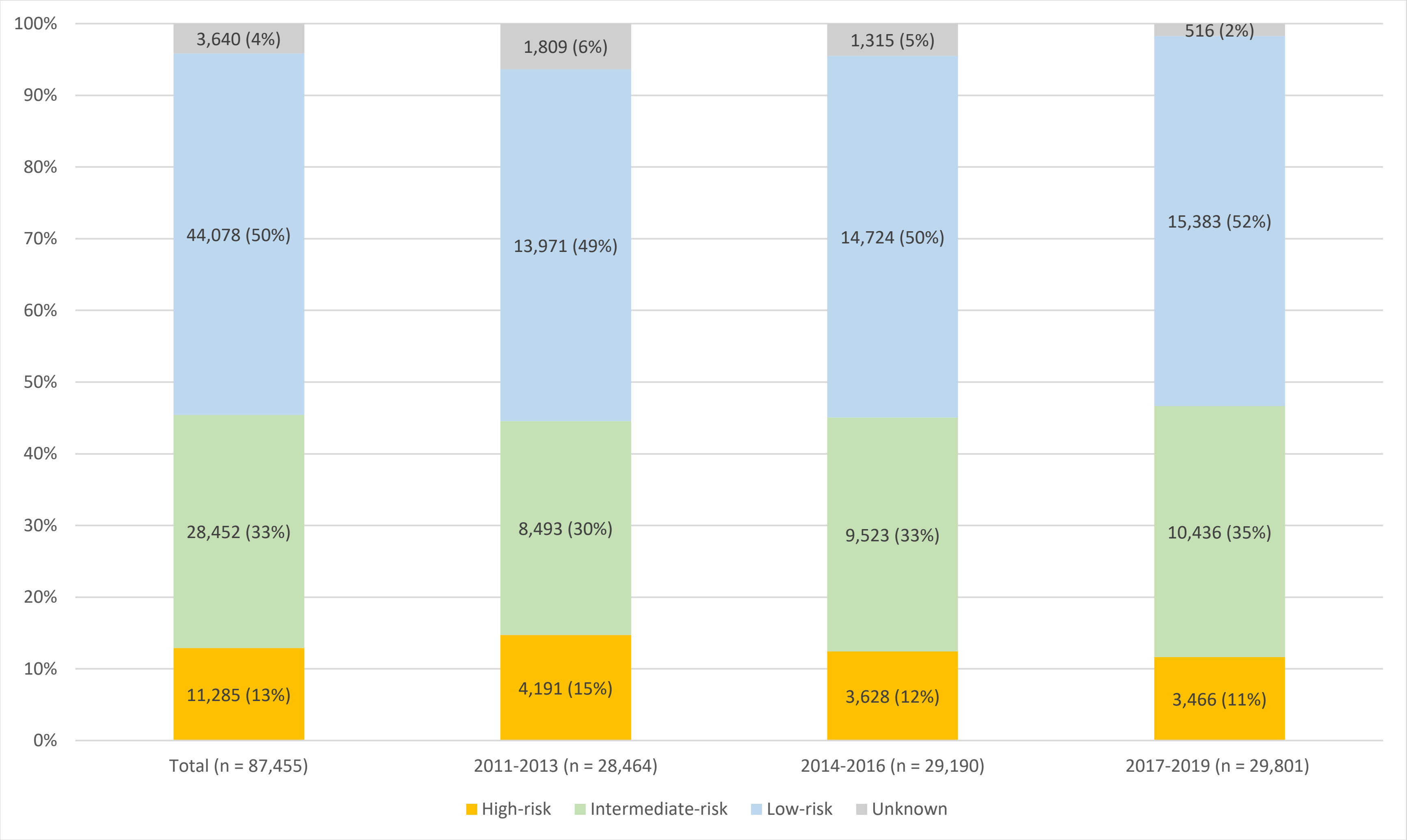

Supplementary Table 3. Baseline characteristics of patients diagnosed with high-risk HR+/HER2- breast cancer by period of diagnosis (N (%))

	**2011-2013 (n = 4,191)**	**2014-2016 (n = 3,628)**	**2017-2019 (n = 3,466)**	**P for trend**
Median age, years (range)	58 (24-94)	57 (20-95)	58 (23-92)	0.46
Age at diagnosis				0.67
<40 years	247 (6)	249 (7)	238 (7)	
40 – 49 years	1,002 (24)	831 (23)	785 (23)	
50 – 75 years	2,386 (57)	2,107 (58)	1,981 (57)	
>75 years	556 (13)	441 (12)	462 (13)	
Tumour size				<0.001
≤2 cm	1,130 (27)	832 (23)	728 (21)	
2.1-4.9 cm	1,907 (46)	1,514 (42)	1,354 (39)	
≥5 cm	1,138 (27)	1,274 (35)	1,380 (40)	
Unknown	16	8	4	
Lymph nodes				<0.001
Negative	0 (0)	0 (0)	0 (0)	
1-3 positive	2,007 (48)	2,155 (59)	2,292 (66)	
4-9 positive	1,453 (35)	988 (27)	794 (23)	
≥10 positive	731 (17)	485 (13)	380 (11)	
TNM stage[a]				0.003
Stage I	181 (4)	178 (5)	153 (4)	
Stage II	1,228 (29)	1,158 (32)	1,142 (33)	
Stage III	2,782 (66)	2,292 (63)	2,171 (63)	
Histological grade				<0.001
Grade 1	240 (7)	279 (9)	259 (8)	
Grade 2	1,297 (36)	1,253 (38)	1,401 (42)	
Grade 3	2,040 (57)	1,742 (53)	1,704 (51)	
Unknown	614	354	102	
Hormone receptor status				0.47
ER+/PR+	3,284 (79)	2,851 (79)	2,751 (79)	
ER+/PR-	868 (21)	732 (20)	692 (20)	
ER-/PR+	26 (1)	45 (1)	22 (1)	
Unknown ER or PR status	13	0	1	
Histology				<0.001
Ductal	3,144 (75)	2,652 (73)	2,480 (72)	
Lobular	942 (23)	879 (24)	884 (26)	
Other	105 (3)	97 (3)	102 (3)	
Axillary staging				
SNP	2,112 (50)	1,961 (54)	2,095 (60)	<0.001
MARI	0 (0)	228 (6)	776 (22)	<0.001
ALND	3,429 (82)	2,113 (58)	1,574 (45)	<0.001
None	48 (1)	85 (2)	44 (1)	0.50
Breast-conserving surgery				0.002
Yes	1,337 (32)	1,210 (33)	1,224 (35)	
Radiotherapy				<0.001
Yes	3,413 (81)	3,196 (88)	3,098 (89)	

Abbreviations: ALND = axillary lymph node dissection; ER = oestrogen receptor; MARI = marking axillary lymph nodes with radioactive iodine ^{125}I seeds; PR = progesterone receptor; SNP = sentinel node procedure; TNM = tumour node metastasis.

Percentages may not add up to 100% because of rounding.

[a]Tumours were categorised according to the TNM classification of malignant tumours which was valid at the time of diagnosis, since only minor differences were present between the 7th or 8th edition. The pathological TNM stage was used when available. In patients with unknown pathological TNM stage as well as patients who received neoadjuvant systemic therapy, the clinical TNM stage was used.

Supplementary Table 4. Systemic treatment choices in patients diagnosed with high-risk HR+/HER2- breast cancer by period of diagnosis (N (%))

**Systemic treatment**	**2011 – 2013 (n = 4,191)**	**2014 – 2016 (n = 3,628)**	**2017 – 2019 (n = 3,466)**	**P for trend**
Endocrine therapy	3,947 (94)	3,423 (94)	3,214 (93)	0.01
Neoadjuvant	111 (3)	174 (5)	314 (9)	<0.001
Adjuvant	3,945 (94)	3,419 (94)	3,204 (92)	0.004
Chemotherapy	3,106 (74)	2,677 (74)	2,439 (70)	<0.001
Neoadjuvant	828 (20)	1,258 (35)	1,458 (42)	<0.001
Adjuvant	2,346 (56)	1,469 (41)	1,170 (34)	<0.001
Endocrine therapy and chemotherapy	2,969 (71)	2,547 (70)	2,296 (66)	<0.001
No systemic therapy	107 (3)	75 (2)	109 (3)	0.13

Supplementary Figure 3. Overall survival (A) and relative survival (B) of all patients diagnosed with and surgically treated for non-metastatic invasive HR+/HER2- breast cancer from date of diagnosis, per 3-year incidence period

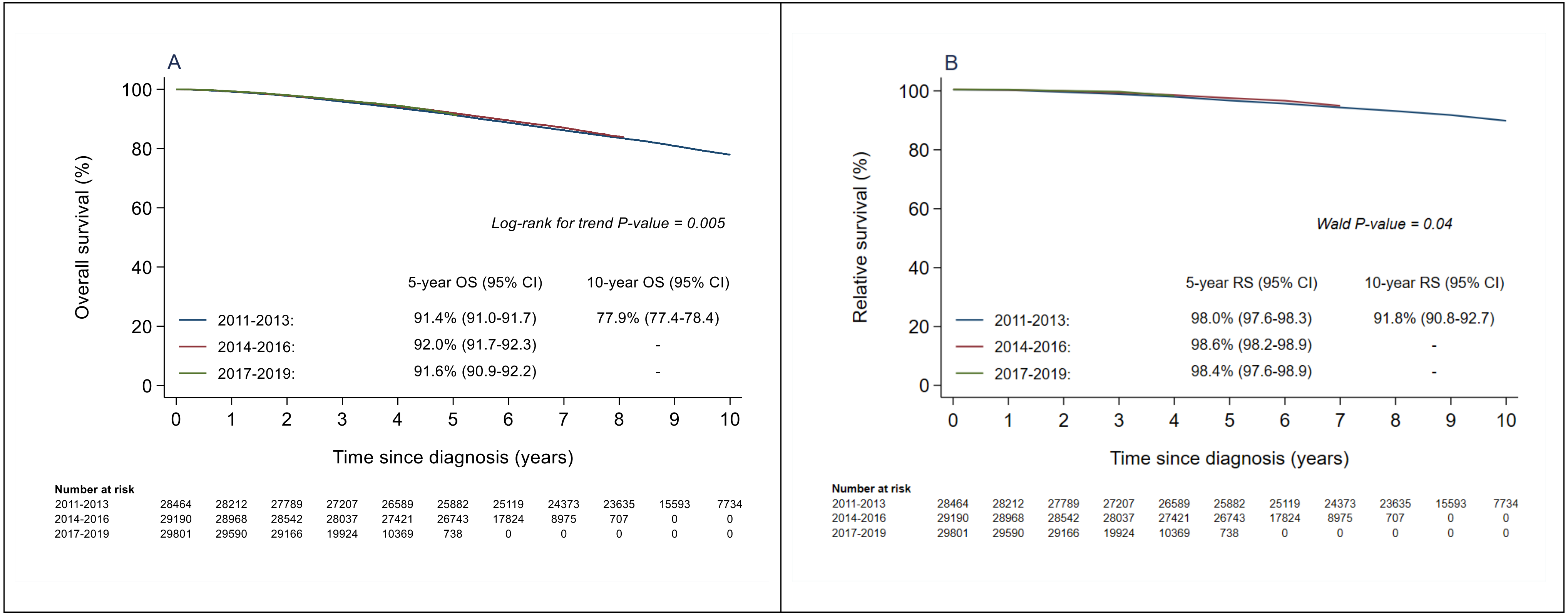

Supplementary Table 5. Univariable and multivariable analyses of overall survival in all patients diagnosed with HR+/HER2- breast cancer

	Univariable analyses		Multivariable analyses	
	HR (95% CI)	P-value	HR (95% CI)	P-value
Period of diagnosis				
2014 – 2016 vs 2011 – 2013	0.95 (0.90 – 0.99)	0.01	0.95 (0.91 – 1.00)	0.03
2017 – 2019 vs 2011 – 2013	0.93 (0.87 – 0.99)	0.02	0.90 (0.84 – 0.96)	0.001
Age				
≥60 years vs <60 years	3.12 (2.98 – 3.27)	<0.001	2.56 (2.44 – 2.69)	<0.001
Tumour size				
2.1 – 4.9 cm vs ≤2 cm	2.09 (2.00 – 2.17)	<0.001	2.19 (2.09 – 2.29)	<0.001
≥5 cm vs ≤2 cm	2.72 (2.54 – 2.90)	<0.001	2.92 (2.70 – 3.15)	<0.001
Number of lymph nodes				
1 – 3 nodes vs no nodes	1.36 (1.30 – 1.41)	<0.001	1.60 (1.52 – 1.67)	<0.001
4 – 9 nodes vs no nodes	2.57 (2.39 – 2.75)	<0.001	2.77 (2.56 – 2.99)	<0.001
≥10 nodes vs no nodes	4.54 (4.19 – 4.91)	<0.001	4.41 (4.04 – 4.81)	<0.001
Histological grade				
Grade 2 vs grade 1	1.38 (1.32 – 1.45)	<0.001	1.35 (1.29 – 1.42)	<0.001
Grade 3 vs grade 1	2.06 (1.95 – 2.17)	<0.001	2.00 (1.88 – 2.13)	<0.001
Hormone receptor status				
Single HR+ vs double HR+	1.44 (1.38 – 1.51)	<0.001	1.27 (1.21 – 1.33)	<0.001
Histology				
(Mixed) lobular vs ductal	1.14 (1.08 – 1.19)	<0.001	0.89 (0.84 – 0.93)	<0.001
Other vs ductal	1.19 (1.10 – 1.28)	<0.001	1.17 (1.08 – 1.27)	<0.001
Chemotherapy				
Yes vs no	0.64 (0.61 – 0.67)	<0.001	0.46 (0.43 – 0.48)	<0.001
Endocrine therapy				
Yes vs no	1.23 (1.18 – 1.28)	<0.001	0.76 (0.72 – 0.80)	<0.001

Abbreviations: CI = confidence interval; HR = hazard ratio; HR+ = hormone receptor-positive.

Supplementary Table 6. Univariable and multivariable analyses of relative survival in all patients diagnosed with HR+/HER2- breast cancer

	**Univariable analyses**		**Multivariable analyses**	
	HR (95% CI)	P-value	HR (95% CI)	P-value
Period of diagnosis				
2014 – 2016 vs 2011 – 2013	0.90 (0.80 – 1.01)	0.06	0.96 (0.87 – 1.06)	0.39
2017 – 2019 vs 2011 – 2013	0.83 (0.68 – 1.01)	0.06	0.94 (0.80 – 1.09)	0.40
Age				
≥60 years vs <60 years	0.83 (0.73 – 0.93)	0.002	1.02 (0.92 – 1.13)	0.72
Tumour size				
2.1 – 4.9 cm vs ≤2 cm	4.44 (3.88 – 5.07)	<0.001	2.80 (2.46 – 3.20)	<0.001
≥5 cm vs ≤2 cm	9.40 (8.11 – 10.91)	<0.001	4.26 (3.65 – 4.98)	<0.001
Number of lymph nodes				
1 – 3 nodes vs no nodes	3.55 (3.07 – 4.09)	<0.001	2.93 (2.56 – 3.36)	<0.001
4 – 9 nodes vs no nodes	10.53 (8.99 – 12.34)	<0.001	6.31 (5.38 – 7.42)	<0.001
≥10 nodes vs no nodes	21.90 (18.68 – 25.69)	<0.001	12.01 (10.22 – 14.11)	<0.001
Histological grade				
Grade 2 vs grade 1	3.48 (2.72 – 4.46)	<0.001	2.29 (1.89 – 2.79)	<0.001
Grade 3 vs grade 1	8.22 (6.41 – 10.53)	<0.001	4.16 (3.40 – 5.08)	<0.001
Hormone receptor status				
Single HR+ vs double HR+	2.23 (2.02 – 2.47)	<0.001	2.02 (1.84 – 2.21)	<0.001
Histology				
(Mixed) lobular vs ductal	1.17 (1.04 – 1.33)	0.01	0.92 (0.82 – 1.03)	0.14
Other vs ductal	0.58 (0.41 – 0.81)	0.001	0.87 (0.66 – 1.14)	0.30
Chemotherapy				
Yes vs no	3.26 (2.84 – 3.74)	<0.001	0.55 (0.49 – 0.61)	<0.001
Endocrine therapy				
Yes vs no	3.10 (2.60 – 3.68)	<0.001	0.69 (0.60 – 0.80)	<0.001

Abbreviations: CI = confidence interval; HR = hazard ratio; HR+ = hormone receptor-positive.

Supplementary Figure 4. Overall survival (A) and relative survival (B) of patients diagnosed with high-risk HR+/HER2- breast cancer from date of diagnosis, per 3-year incidence period

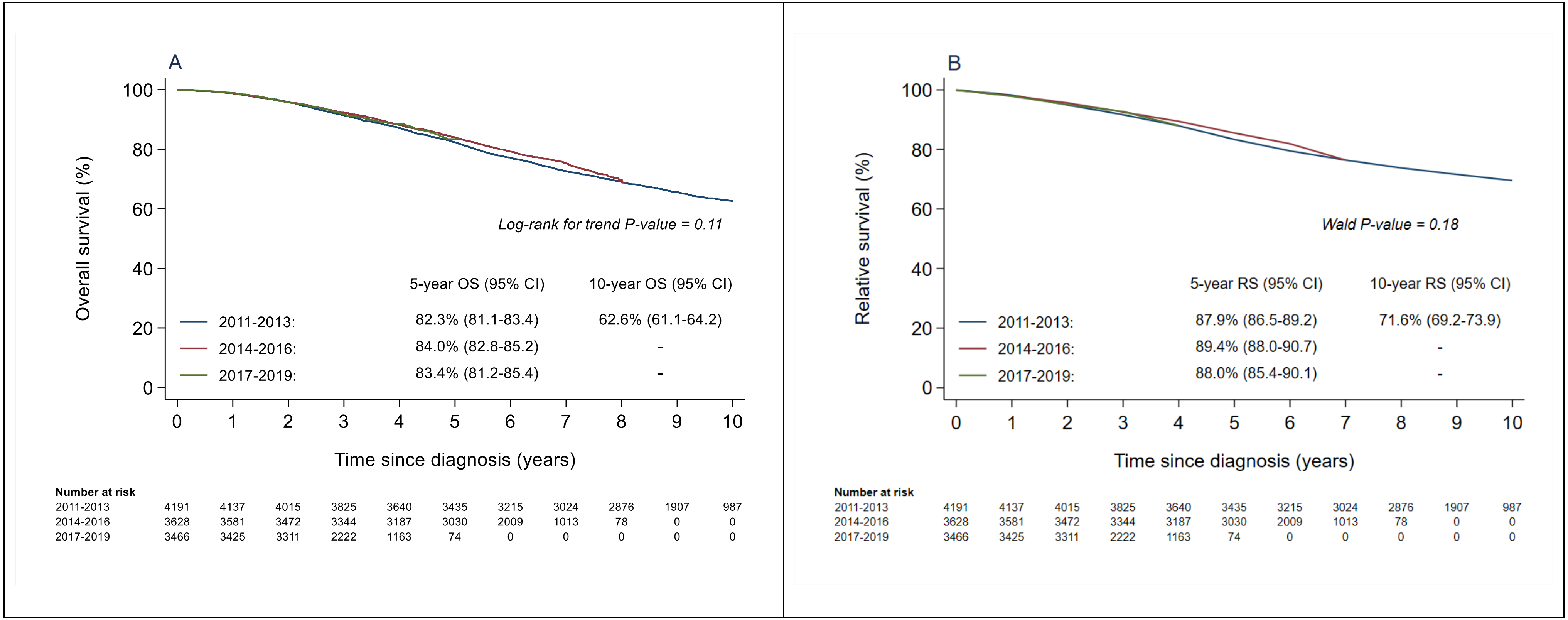

Supplementary Table 7. Univariable and multivariable analyses of overall survival in patients diagnosed with high-risk HR+/HER2- breast cancer

	Univariable analyses		Multivariable analyses	
	HR (95% CI)	P-value	HR (95% CI)	P-value
Period of diagnosis				
2014 – 2016 vs 2011 – 2013	0.92 (0.84 – 1.01)	0.07	0.97 (0.89 – 1.06)	0.48
2017 – 2019 vs 2011 – 2013	0.93 (0.82 – 1.07)	0.28	0.96 (0.85 – 1.09)	0.52
Age				
≥60 years vs <60 years	2.63 (2.43 – 2.85)	<0.001	1.55 (1.41 – 1.71)	<0.001
Tumour size				
2.1 – 4.9 cm vs ≤2 cm	1.83 (1.65 – 2.04)	<0.001	1.61 (1.45 – 1.79)	<0.001
≥5 cm vs ≤2 cm	1.64 (1.47 – 1.84)	<0.001	2.15 (1.91 – 2.42)	<0.001
Number of lymph nodes				
4 – 9 nodes vs 1 – 3 nodes	1.36 (1.25 – 1.49)	<0.001	1.80 (1.63 – 1.98)	<0.001
≥10 nodes vs 1 – 3 nodes	2.39 (2.17 – 2.63)	<0.001	2.86 (2.59 – 3.17)	<0.001
Histological grade				
Grade 2 vs grade 1	1.43 (1.21 – 1.69)	<0.001	1.27 (1.08 – 1.50)	0.005
Grade 3 vs grade 1	1.44 (1.22 – 1.69)	<0.001	1.85 (1.56 – 2.19)	<0.001
Hormone receptor status				
Single HR+ vs double HR+	1.63 (1.50 – 1.77)	<0.001	1.55 (1.42 – 1.68)	<0.001
Histology				
(Mixed) lobular vs ductal	1.16 (1.06 – 1.26)	0.001	0.98 (0.89 – 1.08)	0.67
Other vs ductal	1.04 (0.83 – 1.32)	0.72	0.98 (0.77 – 1.24)	0.86
Chemotherapy				
Yes vs no	0.31 (0.29 – 0.33)	<0.001	0.38 (0.35 – 0.42)	<0.001
Endocrine therapy				
Yes vs no	0.52 (0.46 – 0.60)	<0.001	0.57 (0.50 – 0.65)	<0.001
Abbreviations: CI = confidence interval; HR = hazard ratio; HR+ = hormone receptor-positive.				

Supplementary Table 8. Univariable and multivariable analyses of relative survival in patients diagnosed with high-risk HR+/HER2- breast cancer

	Univariable analyses		Multivariable analyses	
	HR (95% CI)	P-value	HR (95% CI)	P-value
Period of diagnosis				
2014 – 2016 vs 2011 – 2013	0.90 (0.79 – 1.03)	0.12	0.95 (0.84 – 1.08)	0.45
2017 – 2019 vs 2011 – 2013	0.95 (0.78 – 1.16)	0.63	1.02 (0.85 – 1.21)	0.86
Age				
≥60 years vs <60 years	1.52 (1.36 – 1.70)	<0.001	1.09 (0.96 – 1.23)	0.20
Tumour size				
2.1 – 4.9 cm vs ≤2 cm	2.05 (1.72 – 2.44)	<0.001	1.80 (1.53 – 2.12)	<0.001
≥5 cm vs ≤2 cm	2.12 (1.78 – 2.54)	<0.001	2.64 (2.22 – 3.14)	<0.001
Number of lymph nodes				
4 – 9 nodes vs 1 – 3 nodes	1.55 (1.35 – 1.77)	<0.001	2.04 (1.78 – 2.34)	<0.001
≥10 nodes vs 1 – 3 nodes	3.21 (2.81 – 3.68)	<0.001	3.78 (3.31 – 4.33)	<0.001
Histological grade				
Grade 2 vs grade 1	1.74 (1.32 – 2.31)	<0.001	1.48 (1.15 – 1.90)	0.003
Grade 3 vs grade 1	1.72 (1.30 – 2.27)	<0.001	2.27 (1.76 – 2.93)	<0.001
Hormone receptor status				
Single HR+ vs double HR+	2.03 (1.80 – 2.28)	<0.001	1.91 (1.70 – 2.13)	<0.001
Histology				
(Mixed) lobular vs ductal	1.18 (1.03 – 1.34)	0.02	0.97 (0.85 – 1.11)	0.68
Other vs ductal	0.98 (0.68 – 1.41)	0.91	0.92 (0.65 – 1.29)	0.62
Chemotherapy				
Yes vs no	0.48 (0.43 – 0.54)	<0.001	0.47 (0.41 – 0.54)	<0.001
Endocrine therapy				
Yes vs no	0.54 (0.44 – 0.66)	<0.001	0.56 (0.47 – 0.68)	<0.001

Abbreviations: CI = confidence interval; HR = hazard ratio; HR+ = hormone receptor-positive.
